# Edible Clusteroluminogenic Films Obtained from Starch of Different Botanical Origins for Food Packaging and Quality Management of Frozen Foods

**DOI:** 10.3390/membranes12040437

**Published:** 2022-04-18

**Authors:** Wing-Fu Lai, Wing-Tak Wong

**Affiliations:** 1 Department of Applied Biology and Chemical Technology, Hong Kong Polytechnic University, Hong Kong, China; w.t.wong@polyu.edu.hk; 2 Ciechanover Institute of Precision and Regenerative Medicine, The Chinese University of Hong Kong (Shenzhen), Shenzhen 518172, China

**Keywords:** clusteroluminescence, food packaging, starch, films, quality management

## Abstract

Starch is a naturally occurring material showing high potential for use in food packaging because of its low cost, natural abundance and high biodegradability. Over the years, different starch-based packaging films have been developed, but the impact of botanical sources on film performance has rarely been exploited. Efforts devoted to exploiting the role played by the clusteroluminescence of starch in food packaging are also lacking. This study fills these gaps by comparing the properties of edible starch films generated from different botanical sources (including water chestnuts, maize and potatoes) in food packaging. Such films are produced by solution casting. They are highly homogeneous, with a thickness of 55–65 μm. Variations in the botanical sources of starch have no significant impact on the color parameters (including L*, a* and b*) and morphological features of the films but affect the water vapor permeability, maximum tensile strength and elongation at break. Starch films from water chestnut show the highest percentage of transmittance, whereas those from potatoes are the opaquest. No observable change in the intensity of clusteroluminescence occurs when a packaging bag generated from starch is used to package fresh or frozen chicken breast meat; however, a remarkable decline in the intensity of luminescence is noted when the frozen meat is thawed inside the bag. Our results reveal the impact of starch sources on the performance of starch films in food packaging and demonstrate the possibility of using the clusteroluminescence of starch as an indicator to reveal the state of packaged frozen food.

## 1. Introduction

Starch is a naturally occurring material that is cheap and highly biodegradable [1,2,3]. Over the years, extensive efforts have been adopted to explore the use of starch in food packaging [4,5,6,7,8]. For example, corn starch films incorporated with diverse additives (viz., glycerol and sodium montmorillonite) have been exploited as food packaging films, with the hydrophilicity and tensile strength of the films being able to be tuned by changing the contents of the additives [9]. Biodegradable food packaging films have also been obtained by first blending rice starch extracted from broken grains with fish protein recovered from *Micropogonias furnieri*, followed by the incorporation of oregano essential oil. Such films have been found to inhibit peroxidase activity and have demonstrated the potential to be used for anti-browning packaging in fruits and vegetables [10]. Recently, cassava starch films incorporated with polyphenol-rich rosemary extracts have been adopted to generate active food packaging films with antioxidant properties. The films display UV-blocking capacity, which is positively related to the amount of extracts incorporated into the films [11]. The above examples, along with the long history of use of starch in cooking and food product development [12,13,14], demonstrate the practical potential of starch in food packaging. 

Despite this, starch is a complex material that presents several levels of organization [15,16]. It consists of different amounts of amylose and amylopectin [17,18,19,20,21,22], with the molecular weights of these components varying greatly in starch extracted from different botanical sources [23]. It is, therefore, expected that the properties and performance of starch films obtained from different botanical sources are different. However, to date, the impact of botanical sources on the performance of films in food packaging has rarely been seriously exploited. Meanwhile, due to its properties of clusteroluminescence (in which light at long wavelengths is emitted upon the aggregation of non-conjugated electron-rich units in molecules [24,25,26,27,28]), starch is expected to exhibit changes in the intensity of luminescence upon changes in intermolecular distances, which can be affected by changes in the state of the films. Therefore, it is expected that starch films can serve as more than simply plain food packaging films but can be used for quality management of the food product during food packaging. However, until now the role played by the clusteroluminescence of starch in food packaging has not been investigated. The objective of this study is to fill these gaps by first generating edible starch films using starch obtained from water chestnuts, maize and potatoes, followed by detailed characterization and comparison of different properties of the films. In addition, the clusteroluminescence of the generated films is exploited as an indicator to reveal the status of the packaged frozen food. To the best of our knowledge, this is one of the first studies to report starch films as intrinsically luminescent films for intelligent food packaging. Our work revisits conventional and edible starch films with a new perspective, extending the potential use of such films in food packaging applications. 

## 2. Materials and Methods

### 2.1. Materials

Maize starch (MS) and water chestnut starch (WS) were purchased from Laoyao Co., Ltd. (Nanchang, China) and Yuhua Co., Ltd. (Henan, China), respectively. Potato starch (PS) was purchased from Yuhua Co., Ltd. (Henan, China). 

### 2.2. Preparation of Starch Films

A suspension was made by dispersing 0.8 g of starch in 5 mL of distilled water. The suspension was poured, under magnetic stirring, into 15 mL of distilled water, which was preheated to 80 °C. The resulting suspension was heated additionally at 80 °C for 3 min before it was drop-casted onto the surface of a cleaned glass slide. The glass slide was then kept in vacuum at 40 °C for 10 h until complete evaporation of the solvent. Films generated from MS, WS and PS were designated as FMS, FWS and FPS, respectively.

### 2.3. Determination of Film Thickness 

A handheld digital micrometer (Mitutoyo, Mitutoyo Corporation, Tokyo, Japan) with an accuracy of 0.001 mm was used to measure the thickness of a film at 20 randomly selected locations. An average of the 20 measurements was taken as the thickness of the film sample. 

### 2.4. Fourier-Transform Infrared (FT-IR) Spectroscopy 

The structures of MS, WS, and PS were characterized using FT-IR spectroscopy (Nicolet5700; Thermo Nicolet Company, Waltham, MA, USA) under ambient conditions. Spectra were collected in the range of 700–3900 cm^−1^ with a resolution of 4 cm^−1^ and reported as an average of 16 scans. 

### 2.5. Nuclear Magnetic Resonance (NMR)

^1^H-NMR spectra of starch were recorded with a 400 MHz NMR spectrophotometer (JEOL, Tokyo, Japan). DMSO-d6 was used as a solvent.

### 2.6. Molecular Weight Determination

Molecular weight determination was performed using a high-performance size exclusion chromatography (HPSEC) system (Waters 1525; Milford, MA, USA) equipped with a PLGel 5 μm Mixed-C column (Agilent Technologies, Palo Alto, CA, USA). During analysis, 0.1 g of starch was dissolved in 10 mL of DMSO. Upon filtration through a 0.45 μm polyvinylidene fluoride (PVDF) membrane (Millipore, Bedford, MA, USA), the sample was injected and eluted with DMSO at a flow rate of 1 mL min^−1^ for 25 min. The column temperature was maintained at 35 °C. The eluent was monitored using a refractive index detector (Waters 2414; Milford, MA, USA). 

### 2.7. Amylose Content Determination

The amylose content of starch was determined as previously described [29]. The experiments were performed in triplicate.

### 2.8. Thermogravimetric Analysis (TGA) and Derivative Thermogravimetry (DTG)

TGA and DTG of starch were performed using a Q50 TGA (TA Instruments, New Castle, DE, USA) equipped with platinum pans. The study was conducted in an inert atmosphere of nitrogen in a temperature range from 40 °C to 400 °C. The heating rate was set at 10 °C min^−1^.

### 2.9. Determination of the Tensile Strength 

The tensile strength of films with a rectangular shape (width = 1.5 cm, length = 5 cm) was examined using a tensile tester (M350-10CT; Testometric Co., Ltd., Rochdale, Lancashire, UK). During analysis, the films were subjected to a strain rate of 30 mm min^−1^ until breakage occurred.

### 2.10. Scanning Electron Microscopy (SEM) Analysis

Films were sputter-coated with gold. Their morphological features were observed using a JEOL JSM-6380 (JEOL, Tokyo, Japan) microscope operated at an accelerating voltage of 10 kV.

### 2.11. Characterization of Optical Properties of the Films

Transmittance spectra of FWS, FMS and FPS were obtained in the range of 200–800 nm under ambient conditions using an ultraviolet-visible (UV-Vis) spectrophotometer (Jasco V-560; Jasco Co., Ltd., Tokyo, Japan) equipped with a quartz window plate. A holder in the vertical position was used during measurement. Photoluminescence (PL) spectra of the films in both dry and hydrated states were recorded using an FLS920P fluorescence spectrometer (Edinburgh Instruments Ltd., Livingston, UK). PL spectra were recorded at an excitation wavelength of 365 nm. The color and haze of FWS, FMS and FPS were studied using a chroma and haze meter (CS-700; Hangzhou CHN Spec Technology Co., Ltd., Hangzhou, China). Film color was determined based on the CIELAB color system. The haze, lightness (L^⁎^), redness (a^⁎^) and yellowness (b^⁎^) values of the films were obtained. The experiments were performed in triplicate.

### 2.12. Toxicity Assessment

3T3 fibroblasts and HEK293 were cultured in DMEM supplemented with 10% fetal bovine serum. Cells were seeded in a 96-well plate 24 h before the assay at an initial density of 10,000 cells per well and incubated under a humidified atmosphere of 5% CO_2_ at 37 °C. An appropriate amount of the film was ground using a mortar and pestle and resuspended in the fresh cell culture medium to obtain a suspension with the desired concentration. The suspension was filtered using a 0.45-μm polytetrafluoroethylene (PTFE) filter (Advantec Co., Ltd., Tokyo, Japan). The cell culture medium in each well was replaced with 100 µL of the filtrate. After incubation at 37 °C for 5 h, the filtrate in each well was replaced with the fresh cell culture medium. The CellTiter 96 AQueous non-radioactive cell proliferation assay (MTS assay; Promega Corp., Madison, WI, USA) was performed according to the manufacturer’s instructions to determine cell viability (%) in each well, either immediately or after 24 h of post-treatment incubation. The experiments were performed in triplicate.

### 2.13. Contact Angle Measurement

Sessile drop contact angle analysis of FWS, FMS and FPS was conducted using a video-based contact angle measurement system (OCA20; DataPhysics Instruments GmbH, Filderstadt, Germany) incorporated with a software-controlled dosing-volume weight drop. All measurements were performed using water. 

### 2.14. Determination of Water Vapor Permeability and Erosion Susceptibility

The water vapor permeability of FWS, FMS and FPS was determined as previously reported [30]. To determine the resistance of a film to dissolution, a known mass of a dry film was immersed in distilled water and incubated under ambient conditions. Changes in the mass of the film were determined at regular time intervals. The experiments were performed in triplicate.

### 2.15. Evaluation of Film Performance in Food Packaging

Mature Gala apples and boneless, skinless chicken breasts were obtained from local stores (Renrenle, Shenzhen, China). Apples showing no wound signals were used. Each apple was cut into 12 pieces, with the weight of each piece being around 8.5 ± 0.5 g. One apple slice was put into a 50 mL centrifuge tube. A hole with a diameter of 1.5 cm was made on the cap of the tube. The hole was covered with a film affixed to the cap. One tube in which the hole was uncovered was adopted as the control. The tubes were incubated at 4 ± 1 °C. Changes in the weight of the tubes were determined at regular time intervals. The experiments were performed in triplicate.

Boneless, skinless chicken breasts were cut into rectangular pieces, with the surface area of each piece being around 20 cm^2^. A meat cube was either placed directly under ambient conditions or put inside a packaging bag generated from FMS before being placed under ambient conditions. Changes in the weight of the meat cube were determined at regular time intervals. The luminescence of the bags in which meat cubes in different states (viz., fresh meat, frozen meat, and thawed frozen meat) were placed was recorded using a digital camera under UV light irradiation at 365 nm. The experiments were performed in triplicate.

### 2.16. Statistical Analysis

All data were expressed as the means ± standard deviation. Student’s t-test was performed to assess statistical significance. Differences with a *p*-value < 0.05 were considered to be statistically significant.

## 3. Results and Discussion

### 3.1. Structural and Thermal Characterization of Starch of Different Botanical Sources 

**Figure 1:**
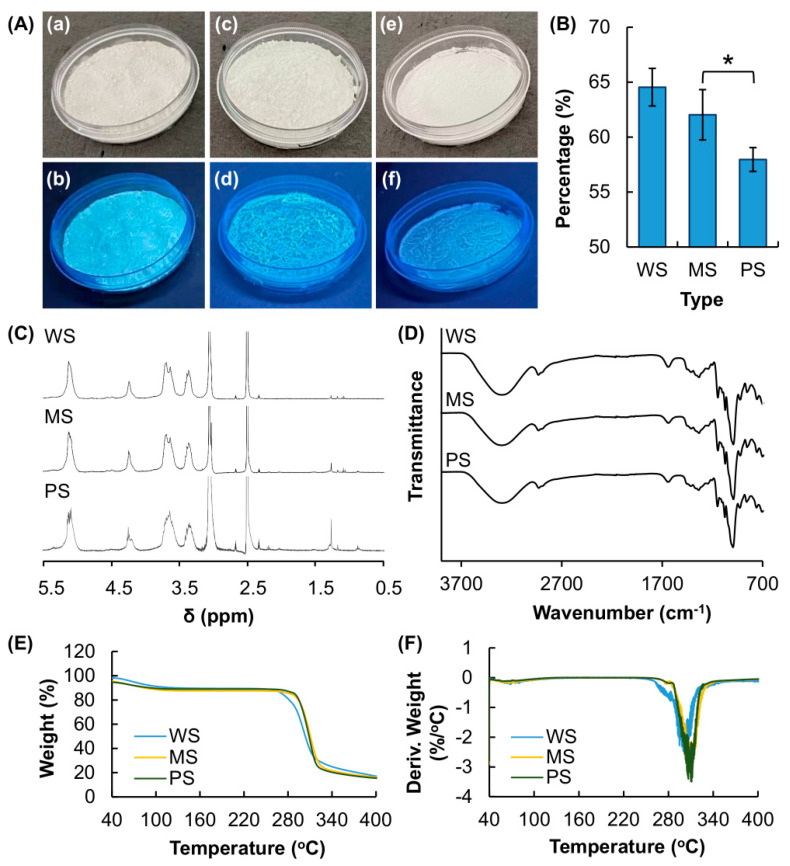
(**A**) Images of (**a**,**b**) water chestnut starch (WS), (**c**,**d**) maize starch (MS) and (**e**,**f**) potato starch (PS) under (**a**,**c**,**e**) white light and (**b**,**d**,**f**) UV light. The wavelength of UV light is at 365 nm. (**B**) Amylose content of starch obtained from different botanical sources. * *p* < 0.05. (**C**) ^1^H-NMR spectra (400 MHz) of WS, MS and PS. The solvent used is DMSO-d6, and the spectra are recorded at 80 °C. (**D**) Fourier-transform infrared (FTIR) spectra, (**E**) thermogravimetric analysis (TGA) curves and (**F**) derivative thermogravimetry (DTG) curves of WS, MS and PS.

Starch is a naturally occurring polymer that exhibits blue luminescence upon excitation at 365 nm (Figure 1A). The mechanism of luminescence is partially attributed to clusterization-triggered emission (CTE) [24,25], during which through-space nonbonding interactions among electron-rich heteroatoms narrow down the energy gap between the HOMO and LUMO to render starch luminescent despite the absence of a conjugated structure. Similar observations on CTE have been reported in other non-conjugated polysaccharides such as cellulose [30,31]. Due to the variations in botanical sources, the content of amylose in WS, MS and PS is different, with the percentage of amylose in PS appearing to be significantly less than that in WS and MS (Figure 1B). 

Despite variations in the amylose content, the NMR spectra of WS, MS and PS show the same pattern (Figure 1C), with chemical shifts assigned to the methylidyne and methylene protons in the glucose unit found between 3.0 and 4.0 ppm. This suggests that starch extracted from the four plant sources is of the same chemical structure. This observation is consistent with the results of FT-IR spectroscopy (Figure 1D), in which the spectra of all starch samples show a signal at 3300 cm^−1^. This signal is attributed to stretching vibrations of the hydroxyl groups of starch and water, as previously described [32]. The peak at 2918 cm^−1^ is attributed to C-H stretching vibrations. The bands at 1147 and 1046 cm^−1^ are assigned to C-O stretching vibrations of the glucose ring. The peak at 991 cm^−1^ is due to C-O stretching vibrations of the C-O-C bond in the α-1,6-linkage.

The thermal properties of starch samples and the corresponding films were determined using TGA and DTG (Figure 1E,F). The TGA curves of all samples showed a significant weight loss from 40 to 100 °C, owing to the evaporation of moisture from the samples. Another weight-loss step is also observed between 267 and 336 °C. This is caused by the degradation of starch molecules [33]. The DTG curves of the samples suggest that the maximum decomposition temperature is around 311 °C. The molecular weights and polydispersity indices (PDIs) of MS, WS and PS are shown in Table 1. There is no significant difference in the molecular weight distribution profiles of the three starch samples adopted. However, the amylose content of starch extracted from different sources is found to be remarkably different, ranging from 64.5 % for WS to 58.0 % for PS. 

### 3.2. Characterization of Physical Properties of the Generated Films

**Figure 2:**
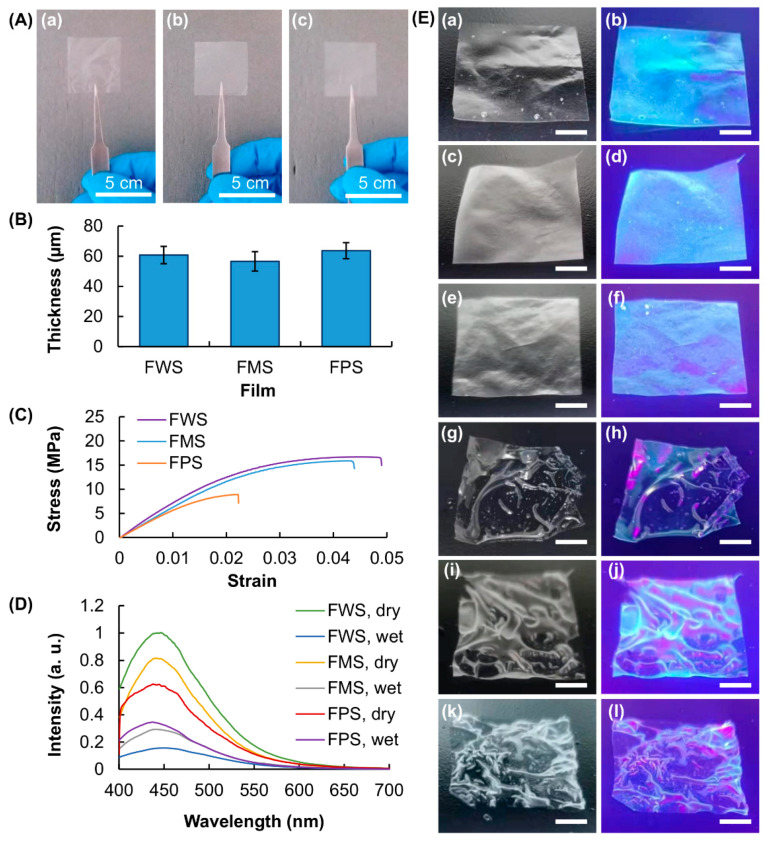
(**A**) Photos of the films generated from (**a**) WS, (**b**) MS and (**c**) PS. (**B**) Thickness and (**C**) tensile strength of the water chestnut starch film (FWS), maize starch film (FMS) and potato starch film (FPS). (**D**) Photoluminescence (PL) spectra of dry and swollen films. (**E**) Images of (**a**,**b**,**g**,**h**) FWS, (**c**,**d**,**i**,**j**) FMS and (**e**,**f**,**k**,**l**) FPS under (**a**,**c**,**e**,**g**,**i**,**k**) white light and (**b**,**d**,**f**,**h**,**j**,**l**) UV light. The wavelength of UV light is at 365 nm. Scale bar = 1 cm.

Solution casting was adopted to generate edible films from WS, MS and PS. The generated films are highly homogeneous, with thickness ranging from 55 to 65 μm (Figure 2A,B). The maximum tensile strength and elongation at break of the generated films are influenced largely by botanical sources of starch, with FWS showing the highest mechanical strength and FPS being the weakest mechanically (Figure 2C). The positive relationship between the amylose content of starch used for film generation and the maximum tensile strength (and elongation at break) of the generated films can be explained by the fact that an increase in the amylose content leads to an increase in the mechanical properties of the gels formed [34,35,36]. This was revealed in an earlier study in which the authors found that the rigidity of a gel formed from starch increases as the amylose: amylopectin ratio increases [34]. The increase in gel strength leads to the formation of a mechanically stronger film, which is generated upon dehydration of the gel. In addition, the intensity of clusteroluminescence varies significantly among FWS, FMS and FPS (Figure 2D,E). This is because starch of different botanical sources varies in the molecular weight and amylose content. This causes variations in the process of through-space non-bonding interactions among electron-rich heteroatoms during aggregation, leading to changes in the intensity of luminescence. In addition, upon hydration, the intensity of luminescence exhibited by all films remarkably drops. This is attributed to the swelling of the films. The process of swelling increases the intermolecular distance of starch molecules, reducing the extent of aggregation of non-conjugated, electron-rich units and leading to a decline in the intensity of CTE. A similar observation has been made in other clusteroluminogenic films [30,31], with the intensity of luminescence of which having been reported to be reduced upon film swelling.

**Figure 3:**
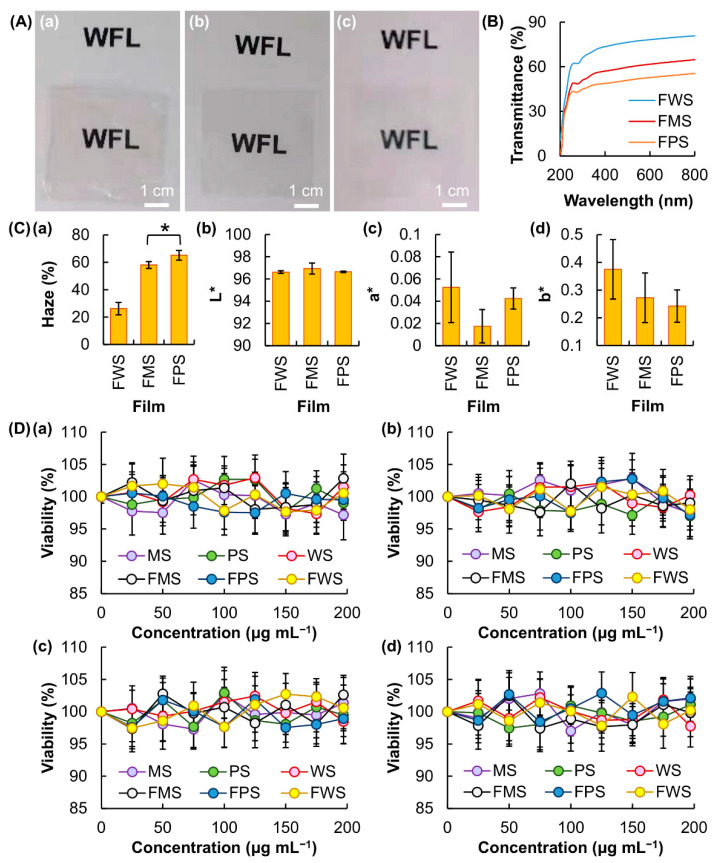
(**A**) Optical images showing the transparency of (**a**) FWS, (**b**) FMS and (**c**) FPS. (**B**) UV-Vis transmittance spectra of different films. (**C**) (**a**) Haze values, (**b**) lightness values (L*), (**c**) redness/greenness values (a*) and (**d**) yellowness/blueness values (b*) of different films. * *p* < 0.05. (**D**) Viability of (**a**,**b**) HEK293 and (**c**,**d**) 3T3 fibroblasts after 5 h of treatment with different samples (viz., WS, MS, PS, FWS, FMS, and FPS) (**a**,**c**) before and (**b**,**d**) after 24 h of post-treatment incubation.

Opacity is an important property of food packaging films because it, on one hand, affects the degree of consumer acceptance and, on the other hand, may contribute to protection of sensitive food components from degradation by absorbing or reflecting a fraction of the incident light to slow down degradation reactions [37]. Although opacity varies among films generated from starch of different botanical sources (Figure 3A), all of the generated films are optically transparent in the visible range (400–700 nm), with the percentage of transmittance ranging from 50% to 80% (Figure 3B). FWS is found to show the highest percentage of transmittance, whereas FPS is the opaquest one. This trend is consistent with the trend in haze (Figure 3C), which is estimated to be around 26% for FWS, 58% for FMS and 65% for FPS. The general appearance and consumer acceptance of packaged food products can be affected not only by the opacity of the films but also by the packaging film color. The lightness values (L*), redness/greenness values (a*) and yellowness/blueness values (b*) are tested and found to have no significant difference among the three generated films (Figure 3C). This suggests that variations in the botanical source of starch does not have a significant impact on the lightness and color of the generated film. 

For a film to be used as a direct food contact material during food packaging, it must demonstrate a lack of toxicity [38]. The toxicity of WS, MS, PS and the generated films (viz., FWS, FMS and FPS) is examined in 3T3 and HEK293 cells. The loss of cell viability after 5 h of treatment with the films is negligible at all concentrations tested (Figure 3D). To determine the possible occurrence of chronic toxicity after treatment, the viability of the treated cells is evaluated after 24 h of post-treatment incubation [39,40,41,42,43]. Our results show that WS, MS, PS and the generated films are lack of both acute and chronic cytotoxicity. This finding suggests that variations in botanical sources have no significant impact on the cytotoxicity of starch and that no change in the toxicity of starch occurs during the process of film fabrication.

### 3.3. Performance as Edible and Indicating Films in Food Packaging

**Figure 4:**
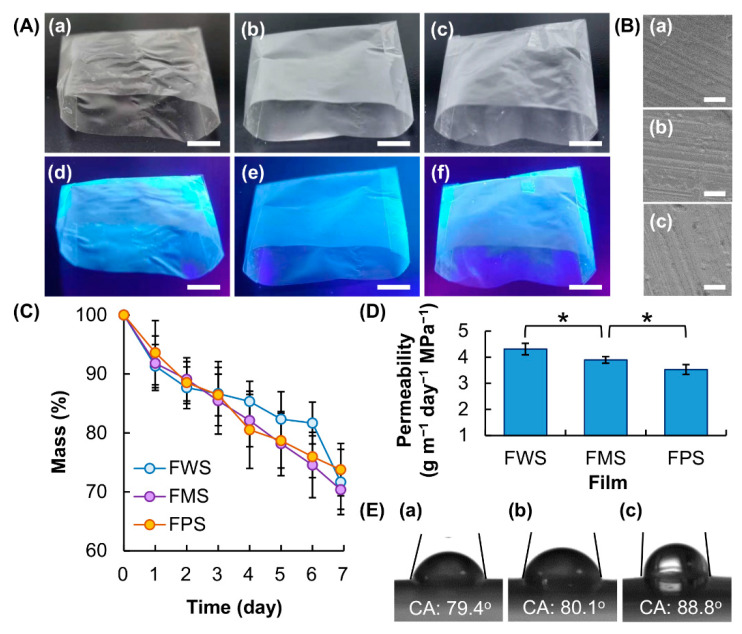
(**A**) Photos of packaging bags generated from (**a**,**d**) FWS, (**b**,**e**) FMS and (**c**,**f**) FPS under (**a**–**c**) white light and (**d**–**f**) UV light. The wavelength of UV light is at 365 nm. Scale bar = 1 cm. (**B**) Scanning electron microscopy (SEM) images of the morphology of (**a**) FWS, (**b**) FMS and (**c**) FPS. Scale bar = 10 μm. (**C**) Erosion susceptibility and (**D**) water vapor permeability of different films. * *p* < 0.05. (E) Measurement of the static contact angle of a water droplet on (**a**) FWS, (**b**) FMS and (**c**) FPS.

The generated starch films are used to produce packaging bags, which exhibit blue luminescence upon excitation at 365 nm due to the process of CTE, for food packaging (Figure 4A). Variations in the botanical origins of starch have no significant impact on the morphology and erosion rate of the generated films (Figure 4B,C); however, starch films generated from different botanical sources differ in their water vapor permeability, which increases in the following order: FPS < FMS < FWS (Figure 4D). The latter may be partially explained by the observed trend in hydrophobicity of the films (FPS > FCS > FMS) as demonstrated by contact angle measurements (Figure 4E). However, this trend is contradictory to what is expected simply based on the amylose content of the starch samples. This is because amylose is less soluble than amylopectin in cold water. Films with a higher amylose content are expected to be more hydrophobic and show lower water vapor permeability. Such a discrepancy is possibly due to, in addition to the amylose content, other structural features (including the molecular weight and degree of polydispersity of amylose and amylopectin, the degree of branching of amylopectin, and the roughness of the film surface) that contribute to determining film properties [3,32,37]. Therefore, simply using the mass percentage of amylose fails to predict the hydrophobicity and water vapor permeability of the generated films.

**Figure 5:**
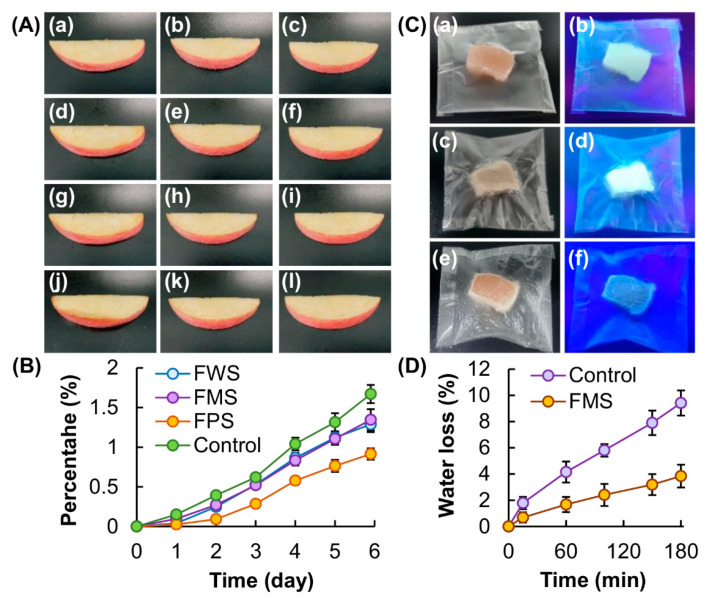
(**A**) Photos of an apple piece stored in a tube with the hole either (**a**–**c**) uncovered or (**d**–**l**) protected by different films ((**d**–**f**) FWS, (**g**–**i**) FMS and (**j**–**l**) FPS) at 4 °C for (**a**,**d**,**g**,**j**) 0 days, (**b**,**e**,**h**,**k**) 3 days and (**c**,**f**,**i**,**l**) 6 days. (**B**) Changes in the weight of an apple piece stored in a tube with the hole either uncovered (control) or protected by different films. (**C**) Photos of a starch-based packaging bag containing (**a**,**b**) fresh chicken meat, (**c**,**d**) frozen chicken meat and (**e**,**f**) thawed frozen chicken meat under (**a**,**c**,**e**) white light and (**b**,**d**,**f**) UV light. (**D**) Time-dependent changes in the water content of the meat stored inside an FMS-generated packaging bag. Meat stored in open air is used as a control.

To demonstrate the performance of the films in protecting packaged food from moisture loss, apple pieces are adopted as a food model (Figure 5A,B). The apple piece in the control group shows the highest level of moisture loss. Compared to the apple pieces protected by FWS and FMS, the one protected by FPS shows the lowest degree of dehydration. This is attributed partly to the low water vapor permeability of FPS, leading to high efficiency in serving as a barrier towards the permeation of water molecules. To exploit the use of CTE of the generated films in food packaging and considering the fact that FMS shows a balance between water vapor permeability and luminescence intensity, we use the FMS-generated packaging bag to package frozen chicken breasts, whose eating quality is susceptible to repeated freeze–thaw cycles during transportation and processing [44,45]. No observable change in the intensity of CTE is noted when the packaging bag is used to package fresh or frozen chicken breast meat (Figure 5C). However, when the frozen meat is thawed inside the bag, the exudate released from the meat leads to hydration (and hence swelling) of the bag. This causes a remarkable decline in the intensity of luminescence. This result reveals the potential of CTE from our starch-based packaging bag to serve as an indicator to reveal the state of the frozen food packaged inside. Apart from this, loss of moisture from the meat is greatly reduced when the meat is put inside the packaging bag (Figure 5D). Along with the edibility of starch, our packaging bag shows high potential to serve as an intelligent and edible device to package food products (e.g., ready-to-bake frozen chapaties [46], frozen fish fillets [47] and frozen beef [48]) whose sensory properties are affected by freeze–thaw cycles. 

## 4. Conclusions

Starch exhibits high biodegradability and natural abundance, showing the potential to overcome some of the problems (including non-degradability and health hazards) caused by synthetic plastics in food packaging. In this study, we compare the properties of edible starch films generated from different botanical sources (including water chestnuts, maize and potatoes) in food packaging. All tested films are optically transparent in the visible range (400–700 nm), with the percentage of transmittance ranging from 50% to 80%. Variations in the botanical sources of starch lead to changes in diverse film properties (including mechanical strength, transparency, swelling capacity and water vapor permeability), although the impact on color parameters and morphological features of the films is not apparent. The ability of our films to reduce moisture loss of packaged food was confirmed by using apple slices and chicken breast meat as food models. In addition, by taking advantage of the moisture-mediated change in the intensity of clusteroluminescence exhibited by the generated films, we demonstrated the potential of the edible starch film to reveal whether packaged frozen meat has undergone freeze–thaw cycles during storage and transportation. Not only do our results reveal the impact of starch sources on the performance of starch films in food packaging; they also provide a new perspective on the use of starch films in food packaging, extending the application potential of such films from mere food protection as demonstrated in conventional studies [49,50,51,52,53] to multifunctional edible food packaging with indicating properties for quality management of packaged frozen food.

## Figures and Tables

**Table 1:** Molecular weights and polydispersity indices (PDIs) of different starch samples.

Sample	Mn (Da)	Mw (Da)	Mp (Da)	Mz (Da)	Mz + 1 (Da)	PDI	Mz/Mw	Mz + 1/Mw
WS	61,534	120,120	92,946	218,567	355,251	1.952097	1.819572	2.957467
MS	77,835	141,681	119,804	223,719	311,611	1.820281	1.579039	2.199390
PS	72,787	144,307	106,446	267,456	430,320	1.982601	1.853384	2.981977

## Data Availability

Data are contained within the article.
